# Robust Visibility Surface Determination in Object Space via Plücker Coordinates

**DOI:** 10.3390/jimaging7060096

**Published:** 2021-06-03

**Authors:** Alessandro Rossi, Marco Barbiero, Paolo Scremin, Ruggero Carli

**Affiliations:** Department of Information Engineering, University of Padova, 35131 Padova, Italy; marco.barbiero.3@studenti.unipd.it (M.B.); paolo.scremin.1@studenti.unipd.it (P.S.); carlirug@dei.unipd.it (R.C.)

**Keywords:** visible-surface determination, ambient occlusion, Plücker coordinates, computer graphics

## Abstract

Industrial 3D models are usually characterized by a large number of hidden faces and it is very important to simplify them. Visible-surface determination methods provide one of the most common solutions to the visibility problem. This study presents a robust technique to address the global visibility problem in object space that guarantees theoretical convergence to the optimal result. More specifically, we propose a strategy that, in a finite number of steps, determines if each face of the mesh is globally visible or not. The proposed method is based on the use of Plücker coordinates that allows it to provide an efficient way to determine the intersection between a ray and a triangle. This algorithm does not require pre-calculations such as estimating the normal at each face: this implies the resilience to normals orientation. We compared the performance of the proposed algorithm against a state-of-the-art technique. Results showed that our approach is more robust in terms of convergence to the maximum lossless compression.

## 1. Introduction

Nowadays, computer vision plays an increasing role in the industrial robotics area [1]. With the transition to Industry 4.0, robotic systems must be able to work even more independently, without the constant supervision of a human operator. Those systems must be able to see and perform decisions based on what they perceive [2]. For example, consider to grab an object, randomly placed within a box, with a robotic manipulator. Typically, a scanner captures a three-dimensional image of the box and then a matching algorithm compares it with the 3D model of the object to find correspondences. By means of complex algorithms, a compatible robot path is then planned. The problem of those algorithms comes with their complexity which is proportional to mesh dimension, i.e., the number of its triangles. At once, they must be efficient in order to satisfy the imposed cycle time and the best practice to reduce it is by removing unnecessary triangles. Especially in the industrial field, many models are characterised by detailed interiors which are invisible. Most of the faces are hidden inside the model and, therefore, this information is redundant for recognition purposes. In some cases, it can be a considerable part of the total mesh, leading to a waste of system memory and a significant decrease in performance. Actually, this problem arises in heavily based ray tracing applications, such as 3D scanner simulators [3]. In this scenario, an algorithm is essential for reducing the number of triangles without losing useful information. In literature, there exist several different techniques to remove hidden surfaces. They are commonly known as visible-surface determination (VSD) algorithms. There exist two main approaches: image space and object space. The former exploits rasterization rendering techniques to convert objects to pixels. Visibility is decided at each pixel position on the projection plane and, therefore, the complexity and the accuracy depend on the resolution of the view image. Image space techniques quickly provide a result, even if it is bounded to a particular view. In other words, surfaces removed for a viewpoint may be visible from another one. This implies the need to run the algorithm every time the object pose or view changes. These features make those methods suitable for rendering optimizations, e.g., to limit the number of primitives to draw achieving better frame rates. This procedure is handled differently by several algorithms: z-buffering [4], binary space partitioning [5], ray tracing, Warnock [6] and Painter algorithms [7] are the most popular ones. In some scenarios, it is preferable to have a global result, which is view independent, even if at a higher computational cost. Actually, object space methods compare each mesh face with all the others to determine which surfaces, as a whole, are visible. Given the dependence on the number of faces those methods are slower but more precise. Some examples have been proposed in [8,9]. There also exists an hybrid approach that exploits an image space technique with different views to merge the results obtaining an approximated global result [10].

This study presents a robust technique to address the global visibility problem in object space. The aim of this work is to develop an algorithm that guarantees theoretical convergence to the optimal result. In particular, we prove that, with a proper sampling technique, it is possible to correctly classify all the visible faces of a mesh using a finite number of computations. In addition, the proposed method does not require pre-calculations such as estimating the normal at each face, which is necessary information for algorithms relying on image space. These properties make this algorithm suitable for offline evaluation to optimize a 3D model by reducing its size. A typical background of interest of this application is the industrial one where the user needs to recognize an object to plan its manufacturing. Usually, such object does not deform but continuously changes its pose. In this case, it would be enough to pre-process the 3D model once using our method before running recognition algorithms.

Actually, we propose an algorithm based on the definition of ambient occlusion to determine the visibility of 3D model triangles. We exploit the ray tracing technique and, consequently, ray-triangle intersection algorithm. This process is further improved by using a test based on Plücker coordinates instead of the widely known Möller-Trumbore algorithm [11]. Plücker coordinates have already been adopted in exact visibility culling methods [12]: despite this, it is difficult to find a comprehensive mathematical treatment and an algorithm that achieves the optimum in solving the visibility problem. Finally, this approach is numerically tested to validate both the choice of such intersection test and the performance with respect to a state-of-the-art VSD method.

The paper is organized as follows. Section 2 summarizes the most recent works regarding VSD methods and the use of Plücker coordinates to speed up ray-triangle intersection tests. Section 3 shows the problem formulation in detail, focusing on the description of ambient occlusion and Plücker coordinates. Section 5 presents design and software implementation of the algorithm: special emphasis is placed on its limits, highlighting possible future improvements. In Section 6, the proposed solution is tested to analyse compression, quality and performance. The results are also compared to the ones obtained from one of the most widely used VSD methods. Finally, concluding remarks and possible extensions are presented in Section 7.

## 2. Related Works

Visibility computation is crucial in a wide variety of fields like computer graphics, computer vision, robotics, architecture and telecommunication. First visibility estimation algorithms aimed to determine visible lines or surfaces in a synthesized image of a three-dimensional scene. These problems are known as visible line or visible surface determination. There exist many different techniques to address the visibility problems, but we can identify two widespread algorithms: z-buffering for local visible surface determination and ray tracing for computing global visibility. The z-buffering and its modifications dominate the area of real-time rendering, whereas ray tracing is commonly used in the scope of global illumination simulations. Besides these, there is a plethora of algorithms to solve specific visibility problems. Sutherland [7] provides a survey on ten traditional visible surface algorithms. Durand [13] gives a comprehensive multidisciplinary overview of visibility techniques in various research areas. Bittner [14] provides a taxonomy of visibility problems based on the problem domain and provides a broad overview of visibility problems and algorithms in computer graphics grouped by the proposed taxonomy.

The visibility problem that this work aims to solve can be classified as global visibility, i.e., to identify which surfaces are invisible independently from the viewpoint of an observer placed outside the 3D model. The algorithms that address such problem aims to determine a data structure able to yield the information about which parts of the geometry are invisible to an external observer. Developing efficient global visibility algorithms is still an open problem. A notable work in this field is presented by Durand et al. [9], where the authors propose a structure called visibility complex encoding all visibility relations in 3D space. Unfortunately, creating such structure is $O(n^{4}\log n)$, where *n* is the number of polygonal faces in the scene. Therefore, those methods are unfeasible for industrial applications and provide more information of what is really needed for the preprocessing purposes of this work. As explained in Section 3, the approach presented in this paper is based on ambient occlusion computation through ray tracing, to estimate the visibility degree of the various faces to an external observer. Concerning ray tracing computations, several intersection algorithms have been developed over the years. According to Jiménez et al. [15], the most used algorithm to test ray-triangle intersection is the one introduced by Möller and Trumbore [11]. Then, slightly different versions have been proposed mainly aimed at taking advantage of specific hardware accelerations as done by Havel [16]. However, if the intersection point is not required, algorithms based on Plücker coordinates could be faster [17].

## 3. Problem Formulation

In this section we describe the problem we aim to solve. We want to identify the visible faces of a 3D mesh by exploiting the concept of ambient occlusion in object space. As we will explain extensively in Section 4.1, we evaluate the visibility of a certain point on a face in relation to the amount of ambient light hitting that point. Our goal is to ensure theoretical convergence to the optimal result, i.e., all faces identified correctly. Moreover, we do not take any restrictive assumptions. Before going into theoretical and implementation details, it is useful to first introduce the core elements composing a 3D model and to provide a brief overview about the visibility problem.

### 3.1. Representation of 3D Objects

**Figure 1:**
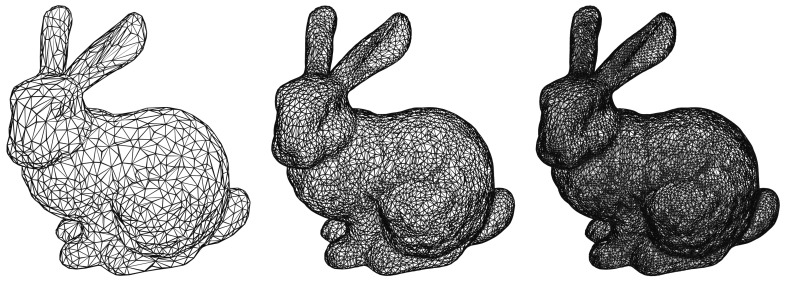
Different approximations of the “Stanford Bunny” using triangular meshes.

There are several ways to represent 3D objects in computer graphics. The most common one consists in considering an object as a collection of surfaces. Given the massive amount of different object types, several surface models have been developed over the years, with an increasing level of complexity according to the level of detail required. Figure 1 shows how a complex surface can be arbitrarily approximated using simple triangles.

The resulting collection of surfaces is called polygonal mesh or simply mesh. Object rendering can be simplified and sped up using polygonal meshes since all surfaces can be described with linear equations. In particular, triangular meshes are preferred for their simplicity, numerical robustness and efficient visualization [18]. Polygonal meshes can be represented in a variety of ways by using the following core elements:

- vertex: a vector representing the position in 3D space along with further information such as colour, normal vector and texture coordinates;
- edge: the segment between two vertices;
- face: a closed set of edges, i.e., a triangle.

**Figure 2:**
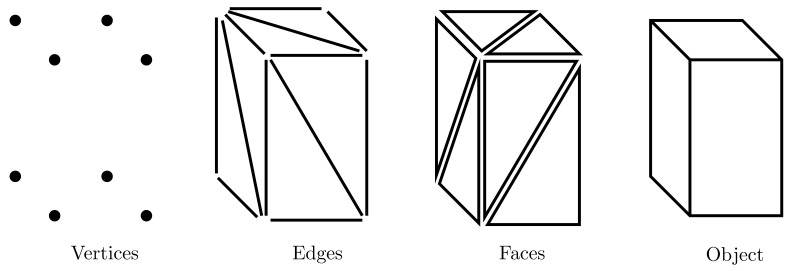
Representation of the core elements of a mesh.

These elements are represented in Figure 2.

This work aims to identify which faces of the 3D model are visible or not. By visible we mean there is at least one point outside the model for which there exists a segment, from that point to another on the triangle, that does not intersect any other.

### 3.2. Visibility Problem

The visibility problem has been one of the first major research topics in computer graphics and is the core of many state-of-the-art algorithms. This concept can be intuitively defined in terms of lines: two points *A* and *B* are mutually visible if no object intersects the line AB between them. From this simple notion, it is possible to address the visibility problem in different spaces regarding where points *A* and *B* lie [13].

- Image space: a 2D space for the visual representation of a scene. The rasterization process of converting a 3D scene into a 2D image works in this space. For this reason, the most common methods to solve the visibility problem perform their operations in 2D projection planes.
- Object space: a 3D space in which the scene is defined and objects lie. Methods developed within this space are computationally onerous and they are usually used to create proper data structure used to sped up subsequent algorithms. These acceleration structures are crucial for real time computer graphics applications such as video games.
- Line space: the one of all the possible lines that could be traced in a scene. Methods developed within this space try to divide the line space according to the geometry that a given line intercepts. Indeed, as stated at the beginning of this section, the visibility notation can be naturally expressed in relation to those elements.
- Viewpoint space: the one of all the possible views of an object. Theoretically, it could be partitioned into different regions divided by visual events. A visual event is a change in the topological appearance. For example, while rotating a coin vertically, at a certain point, one of its faces becomes visible while the other not. This process generates a structure referred in literature as aspect graph [19]. The latter has only a theoretical interest since it could have a $O(n^{9})$ complexity for general non convex polyhedral objects in a 3D viewpoint space, where *n* is the number of object faces. Nevertheless, a few visibility problems are defined and addressed in this space, such as viewpoint optimization for object tracking.

### 3.3. Problem Statement

In this work, the visibility of a certain point on a face is defined in relation to the quantity of ambient light hitting that point; this is the definition of ambient occlusion that we review in Section 4.1. Here, we address the visibility problem in object space and, without loss of generality, we consider triangular meshes. It is trivial to extend the results presented in the following sections to a mesh composed of generic polygons. The problem we want to address can be formally cast as follows. Assume we have a 3D mesh T composed by *N* triangles, i.e., $T=\{T_{1},\ldots ,T_{N}\}$. The triangle $T_{i}$ is said to be visible, if there exist a ray starting from a point on $T_{i}$ and proceeding to infinity that does not intersect any other triangle of the mesh. The goal is to determine, for i=1,…,N, if $T_{i}$ is visible or not.

## 4. Proposed Approach: Ambient Occlusion, Visibility Index and Plücker Coordinates

The approach we propose to deal with the problem stated in Section 3.3 is based on the notion of ambient occlusion. Inspired by this definition, we introduce a visibility index which allows us to estimate if a triangle is visible or not. Basically, given a number of rays starting from points of a triangle, we try to determine if they intersect other triangles of the mesh. We will see that the intersection test can be done in an efficient way exploiting Plücker coordinates and the so-called side operator.

### 4.1. Ambient Occlusion

**Figure 3:**
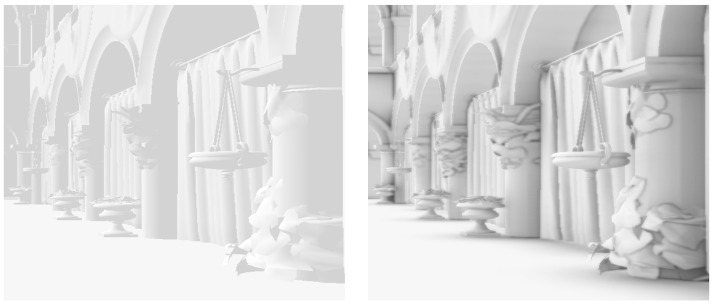
Visual comparison of a scene illuminated by ambient light. The image on the right shows how ambient occlusion estimation increases the realism of the scene. In particular, note that this method generates soft global shadows that contribute to the visual separation of objects.

Ambient occlusion is a rendering technique used to improve the realism of a scene. It is an empirical technique introduced for the first time by Zhukov et al. to approximate the effects produced by global illumination systems [20]. The ambient occlusion models how a certain surface is illuminated by indirect light caused by the reflections of direct light over the various surfaces of the scene. Figure 3 shows the improvements that such technique brings to a rendered image.

The indirect light is approximated by considering an omnipresent, omnidirectional light source with fixed intensity and colour which is called ambient light. Then the exposure of a certain surface to this light is computed by looking at the geometrical properties of its surrounding. In other words, ambient occlusion is an approximation of the darkening which occurs when the ambient light is blocked by nearby objects. Formally, such term is defined for a point on the surface as the cosine-weighted fraction of the upper hemisphere where incoming light cannot reach the point. To be more precise, the ambient occlusion term for a point *P* on a face with normal *n* corresponds to the integral

(1) $$AO\left(P\right)=\frac{1}{\pi }\int_{\omega \in \Omega }V\left(P,\omega \right)\cos\theta d\omega$$

where:

- *P* is the surface point;
- Ω is the upper hemisphere generated by the cutting of a sphere centred in *P* by the plane on which the surface lies;
- ω is a point of the hemisphere Ω and identifies the incoming light direction (with a slight abuse of language, in the following we will sometimes refer to ω as the ray direction);
- V(P,ω) is a function with value 1 if there is incoming ambient light from direction ω and 0 if not;
- $\frac{1}{\pi }$ is a normalization factor;
- θ is the angle between direction ω and the surface normal *n* (note also that cosθ=ω·n).

**Figure 4:**
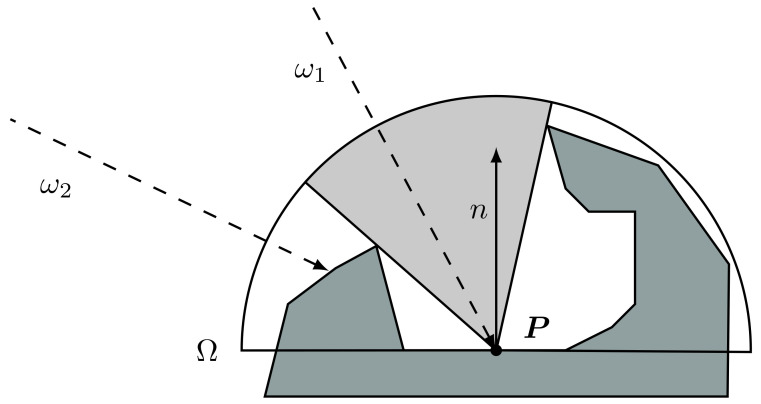
Visual representation of the ambient occlusion for a point *P*. For the rays in example, the function V(P,ω) has the following results: $V(P,\omega_{2})=0$, while $V\left(P,\omega_{i}\right)=1,\;\forall \omega_{i}$ in the grey area of the hemisphere Ω, in particular for $\omega_{1}$.

In Figure 4, a visual representation of the problem is presented.

The integral in (1) is usually evaluated using Monte Carlo techniques by sampling the upper hemisphere in *K* points and, for each one, cast a ray to test for occlusions. Therefore, the integral is approximated by:

(2) $$\hat{AO}\left(P\right)=\frac{1}{K}\sum_{i=0}^{K-1}V\left(P,\omega_{i}\right)$$

with $\omega_{i}$ sampled with probability density function

(3) $$p\left(\omega_{i}\right)=\frac{\cos(\theta_{i})}{\pi }=\frac{\omega_{i}\cdot n}{\pi }\;.$$

Notice that normal *n* shall be oriented correctly towards the outside of the mesh for (3) to be a probability density.

Several different algorithms can be adopted to estimate the ambient occlusion: [21] reviews exhaustively the evolution of ambient occlusion techniques in recent years.

### 4.2. Visibility Index

We describe the strategy used to determine if a point *P* of a triangle of the mesh is visible or not. Suppose *K* rays, say $r_{0},\ldots ,r_{K-1}$, starting from point *P* have been generated. Notice that each ray $r_{i}$ is well identified by its direction $\omega_{i}$. Then, based on the notion of ambient occlusion and, in particular, on its Monte Carlo estimate in Equation (2), the visibility score of a point *P* can be expressed as

(4) $$PV_{score}\left(P\right)=\frac{1}{K}\sum_{i=0}^{K-1}V\left(P,\omega_{i}\right)$$

where V(P,ω)=1 if the ray does not intersect any other triangle and proceed infinitely. Therefore, the point visibility score $PV_{score}$ of a point *P* is the fraction of light-rays which are un-occluded. The choice for this visibility score seems well posed since an observer along the direction of such un-occluded rays is able to see the point *P*. By testing enough points $\left\{P_{1},\ldots ,P_{M}\right\}$ on a face *T* it is possible to estimate its visibility score $SV_{score}$ as the mean of its points scores, giving

(5) $$SV_{score}\left(T\right)=\frac{1}{M}\sum_{i=0}^{M-1}PV_{score}\left(P_{i}\right).$$

After the computation of such score for all the faces of the object it is possible to select only the most visible ones applying a global threshold, effectively removing the internal geometry of the model. Actually, a zero threshold allows to select only the visible faces.

The most costly part of the algorithm is to determine the value of the function V(P,ω). This translates in determining if any given ray intersects any other triangle of the mesh along its path. In this study, this process has been developed using Plücker coordinates and tested against the widely known Möller-Trumbore ray-triangle intersection algorithm. In our implementation every ray is tested against all the other object faces until a first intersection is found.

### 4.3. Plücker Coordinates

Firstly introduced by the mathematician Julius Plücker in 19th century, these coordinates provide an efficient representation of oriented lines. Any real line can be mapped to the Plücker space. Since coordinates are homogeneous, any two points on the same oriented line will have the same Plücker coordinate up to a scaling factor. Let $P=(P_{x},P_{y},P_{z})$ and $Q=(Q_{x},Q_{y},Q_{z})$ be two distinct points in $R^{3}$ which define an oriented line *l* that goes from *Q* to *P*. This line corresponds to a set *l* of six coefficients, called Plücker coordinates of the line *l*

$$l=(l_{0},l_{1},l_{2},l_{3},l_{4},l_{5})$$

where the first three represent the direction of the line. Actually,

$$\begin{array}{c} l_{0}=P_{x}-Q_{x},\;l_{1}=P_{y}-Q_{y},\;l_{2}=P_{z}-Q_{z}, \end{array}$$

while the other three components are given by the cross product between *P* and *Q*, giving

$$\begin{array}{c} l_{3}=P_{y}Q_{z}-P_{z}Q_{y},\;l_{4}=P_{z}Q_{x}-P_{x}Q_{z},\\ l_{5}=P_{x}Q_{y}-P_{y}Q_{x}. \end{array}$$

An important aspect of these coordinates, which has been crucial to this study, is the so-called side operator [22]. Such function characterizes the relative orientation of two lines. Given $l=(l_{0},l_{1},l_{2},l_{3},l_{4},l_{5})$ and $r=(r_{0},r_{1},r_{2},r_{3},r_{4},r_{5})$, the side operator is defined as:

(6) $$\mathrm{side}\left(l,r\right)=l^{T}Wr$$

where

$$W=\left[\begin{array}{cc} 0_{3\times 3} & I_{3\times 3}\\ I_{3\times 3} & 0_{3\times 3} \end{array}\right].$$

Therefore, the side operator can also be written as

(7) $$\mathrm{side}\left(l,r\right)=l_{3}r_{0}+l_{4}r_{1}+l_{5}r_{2}+l_{0}r_{3}+l_{1}r_{4}+l_{2}r_{5}.$$

Two oriented lines *l* and *r* can interact in space in three different ways:

1. if *l* intersects *r*, then side(l,r)=0;
2. if *l* goes clockwise around *r*, then side(l,r)>0;
3. if *l* goes counter-clockwise around *r*, then side(l,r)<0.

**Figure 5:**
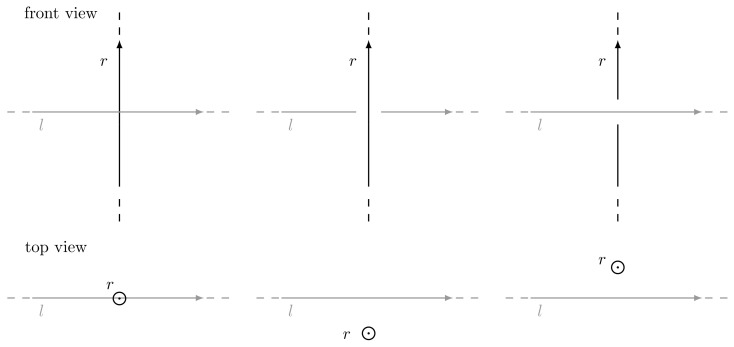
Possible line configurations in space: on the left *l* and *r* intersect, in the middle *l* goes clockwise around *r* while on the right *l* goes counter-clockwise around *r*.

Such cases are highlighted in Figure 5.

Plücker coordinates can be defined in other less intuitive ways and possess other properties that are not used in this work. Also note that not all the Plücker points define a real line in 3D space, but only those that are on the Plücker hypersurface, also known as Grassman manifold or Klein quadric. Such points *l* satisfy the condition side(l,l)=0.

## 5. Visibility Algorithm Based on Plücker Coordinates

**Figure 6:**
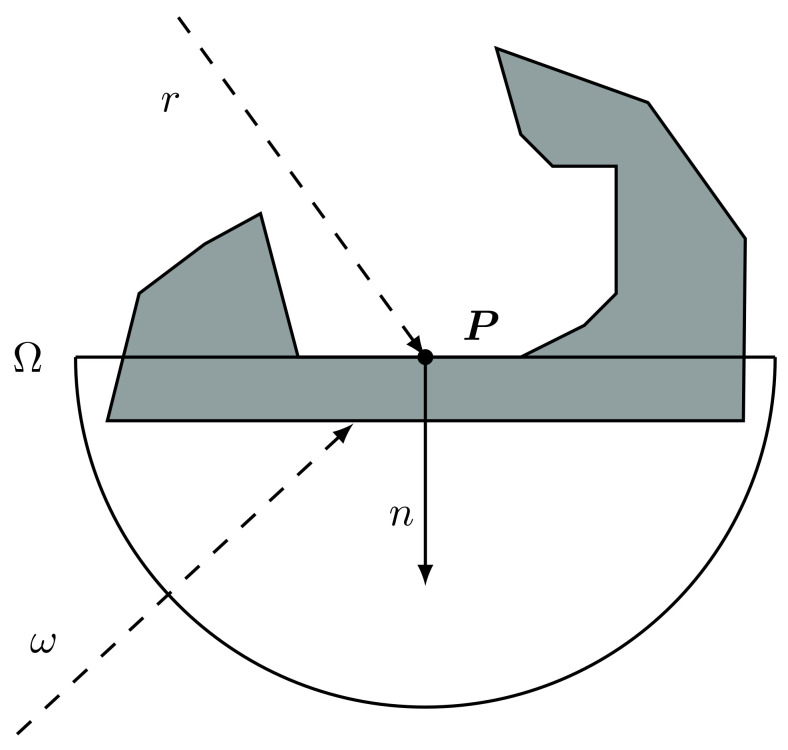
Example of a wrongly oriented normal that results in a false occlusion. By considering only the hemisphere Ω oriented along the normal *n*, the classification gives a wrong result: the ambient occlusion term is zero. Indeed ∀ω∈Ω we have an occlusion but there is at least one un-occluded ray *r* that reaches *P*.

This section starts with an overview of the main steps of the algorithm and, then, describes the implementation details. First, we want to emphasize that typically, in computer graphics, it cannot be assumed that triangles normals are always directed outwards the mesh. Actually, if only the upper hemisphere is considered, like in Figure 4, the procedure may create false occlusions. To avoid such scenario, it is sufficient to consider the whole sphere instead. A false occlusion example is presented in Figure 6.

The algorithm may be divided in three major steps for each mesh triangle $T_{i}$ for which to compute the visibility.

**Initialization:** We select a set of *M* points, say $P=\{P_{1},\ldots ,P_{M}\}$, on $T_{i}$. For each point $P_{i}\in P$, we generate *K* rays as follows: we first create a sphere centred in $P_{i}$, then we sample *K* points on the surface of this sphere and, finally, we draw the *K* rays starting from $P_{i}$ and passing through the previously sampled points. Notice that we generate in total MK rays. How selecting the points on $T_{i}$ and sampling the sphere is described in Section 5.1 and Section 5.2, respectively.

**Score computation:** Each triangle $T_{j}\neq T_{i}$ is tested against all the rays generated at the previous step to see if an intersection occurs. The ray-triangle intersection test is performed in Plücker space as it will be explained in Section 5.3. If a ray intersects a triangle $T_{j}$, then it is removed from the set of generated rays. Once the above intersection testing phase has been completed, the visibility score of $T_{i}$ is computed as

(8) $$SV_{score}\left(T_{i}\right)=\frac{\mathrm{remaining}\;\mathrm{rays}}{MK}$$

**Classification:** If $SV_{score}\left(T_{i}\right)>0$ then $T_{i}$ is classified as visible, otherwise as invisible.

A couple of remarks are now in order.

**Remark** **1.** *Typically, in computer graphics, meshes consist of a large number of triangles that are very small compared to the size of the mesh itself. Based on this fact, in order to reduce the computational burden of the proposed algorithm, in the initialization step we might consider only the barycenter of $T_{i}$, in place of selecting M points; by doing that, we generate only K rays per triangle instead of MK. Our numerical tests show that such choice does not affect the overall result.*

**Remark** **2.** *Note that it is possible to select triangles that are barely visible using a non-zero threshold δ≥0 on the score. Formally, $T_{i}$ is invisible or barely visible if $SV_{score}\left(T_{i}\right)\leq \delta$.*

Next, we explain more in detail how to select the *M* points on a face of the mesh, how to generate *K* rays on a sphere and how to compute the intersections of these rays with mesh triangles.

### 5.1. Sampling Points on Triangle

The points on a triangle $T_{i}$ can be efficiently selected by taking random points at minimum distance or uniformly distributed.

**Uniform distribution:** Arvo [23] presents a procedure for deriving an area-preserving parametrization from a unit square [0,1]×[0,1] to a given bounded 2-manifold $M\subset R^{n}$. Using those results it is possible to uniformly sample any bounded surface, in particular a triangle, by having two independent random variables uniformly distributed over the interval [0,1].

**Minimum distance:** Random samples are generated on a rectangle containing the triangle $T_{i}$ using the Fast Poisson Disk Sampling method [24]. Then, following the rejection-sampling approach, only points that belong to that triangle are kept. The benefit of this algorithm is to apply a constraint on the minimum distance between the generated points, solving the typical clustering issue of uniform sampling methods.

The uniform distribution method is certainly easier to implement and faster to compute, although it tends to generate clusters of points. On the other hand, the minimum distance approach provides a more distributed set since it imposes a constraint on mutual distances. Therefore, to generate a few points, we recommend using the second method.

### 5.2. Rays Generation through Sphere Sampling

A key feature for a VSD algorithm is the ability to uniformly explore the surrounding space. It is, in fact, very important to select points on the surface of a sphere as uniform as possible. The two most common methods to generate uniformly distributed points on a spherical surface are the following two.

**Uniform distribution:** An easy method to uniformly pick random points on an *n*-dimensional sphere is presented in [25]. It is sufficient to generate an *n*-dimension vector $x={\left[x_{1},x_{2},\ldots ,x_{n}\right]}^{T}$ whose $x_{i}$ elements are independent and identically distributed samples of a Gaussian distribution. Then, a point *P* on the sphere is given by $P=\frac{x}{{\left||x|\right|}_{2}}$.

**Fibonacci lattice distribution:** By using the notion of golden ratio and golden angle, both deriving from the Fibonacci sequence, it is possible to generate a set of samples at the same distance from their neighbours [26,27]. In fact, the golden angle optimizes the packing efficiency of elements in spiral lattices.

**Figure 7:**
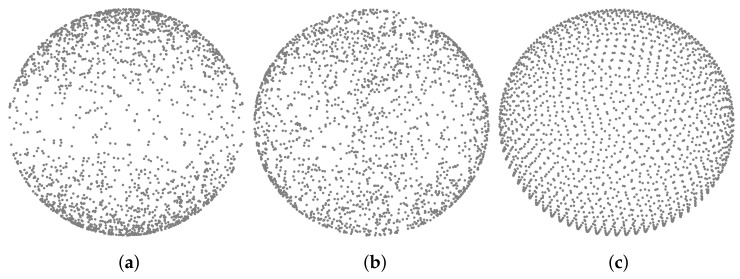
Comparison of three different points distributions on the surface of a sphere: (**a**) Cosine-weighted, (**b**) Uniform, (**c**) Fibonacci.

Applying one of the following algorithms on a unitary sphere centred in the axis origin is equivalent to generate a collection of rays directions. Recalling that the visibility score for a given face $T_{i}$ is defined as the fraction of un-occluded rays over the total rays created, it is important to notice that the choice of one algorithm in place of another will change the value and the meaning of that visibility score. The uniform distribution is certainly easier to implement than Fibonacci’s one but tends to generate clusters when the number of sampled points is small. On the other hand, Fibonacci lattice provides greater uniformity even in the case of a few points. Figure 7 shows the sampling results obtained using the two methods described above. The figure also shows the result obtained with the cosine-weighted distribution [23]. The latter is the only one that allows to correctly estimate the ambient occlusion value as defined is Section 4.1. However, we can observe how this choice is not suitable for our aim since it tends to accumulate points near the poles and, consequently, rays are not well distributed. Notice that we are interested to evaluate the occlusion determined by homogeneously distributed rays rather than mathematically estimate the ambient occlusion term. Therefore, from our tests, we conclude that Fibonacci lattice provides the best distribution for our goal.

### 5.3. Ray Intersection Algorithm via Plücker Coordinates

**Figure 8:**
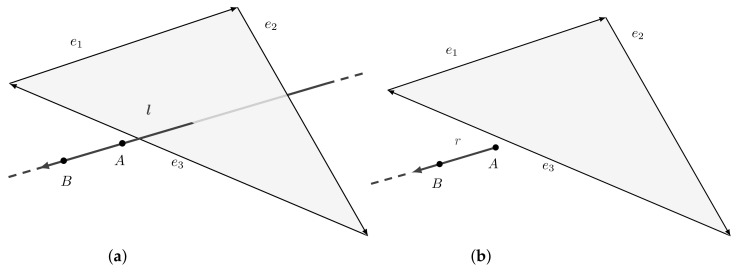
Comparison between line and ray intersection tests. (**a**) Considering the line passing through *A* and *B*, there is an intersection with the polygon. (**b**) Considering the ray starting in *A* and passing through *B*, there is no intersection.

We are now interested to determine the intersection between a ray and a triangle in Plücker space. This is not trivial since, in this space, it is not possible to define rays but only lines. Recall that a line extends infinitely in both directions while a ray is only a portion of it, starting from a point and going to infinity. This implies the inability to directly use a line-polygon intersection test to search for intersections between a ray and a polygon. Figure 8 shows the difference between the results obtained using line and ray intersection tests. In this section, we first describe Plücker line-polygon intersection test and, then, we modify it to support ray-polygon intersection.

**Figure 9:**
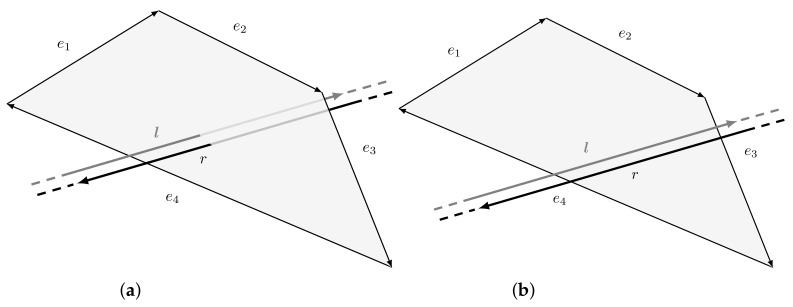
(**a**) Lines stabbing a convex polygon in 3D space. Note that the line l goes counter-clockwise around all the edges $e_{i}$, while r clockwise. (**b**) Lines missing a convex polygon in 3D space. Note that the line l goes counter-clockwise around the edges $e_{1}$ and $e_{4}$, while clockwise around $e_{2}$ and $e_{3}$. The opposite happens with r.

As introduced in Section 4.3, Plücker coordinates can be used to define an efficient line-polygon intersection test thanks to the side operator. Actually, a line hits a polygon if and only if it hits one of the polygon edges, or goes around clockwise or counter-clockwise to all the edges [28]. Figure 9 shows two examples of such test. The convention chosen is to define a polygon as a sequence of vertices $\left\{X_{1},\ldots ,X_{N}\right\}$ in such a way that the direction of the edges $e_{i}$ is defined from $X_{i}$ to $X_{i+1}$; in particular, the last edge goes from $X_{N}$ to $X_{1}$.

Consider the edges $e_{i}$ of a convex polygon with i={1,…,m},m∈N≥3 and a line *l*. The line *l* intersects the polygon if and only if

$$\begin{array}{c} \forall e_{i},\;\mathrm{side}\left(l,e_{i}\right)\geq 0\;\mathrm{OR}\;\forall e_{i},\;\mathrm{side}\left(l,e_{i}\right)\leq 0\;. \end{array}$$

After describing the line-polygon intersection test method, we show how it is possible to use it as a ray intersection test [29,30].

First of all, consider a plane π containing the ray origin *P*. Observe that this plane divides the 3D space in two half-spaces $H_{f}$ and $H_{b}$, where we assume the former contains the ray trajectory. Now, let *n* be the versor orthogonal to π starting from *P* and contained in $H_{f}$. Then, the two half-spaces are formally defined as:

$$\begin{array}{cc} H_{f} & =\{x\in R^{3}:n\cdot \left(x-P\right)>0\}\\ H_{b} & =\{x\in R^{3}:n\cdot \left(x-P\right)<0\}\;. \end{array}$$

**Figure 10:**
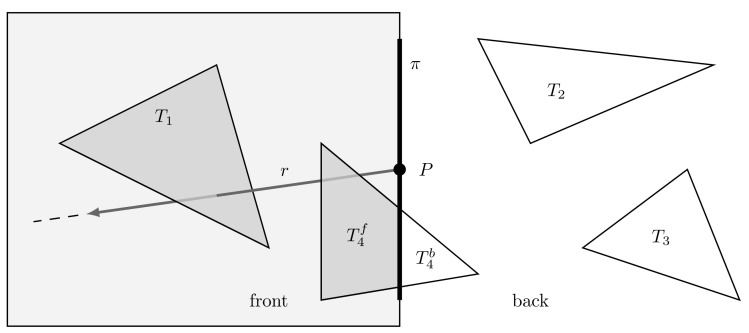
A ray-polygon intersection can be seen as a line-polygon one by considering only front geometries. Notice the case of the polygon $T_{4}$ that overlaps the plane π: in this case a clipping procedure is needed to subdivide it in $T_{4}^{f}$ and $T_{4}^{b}$.

As shown in Figure 10, ray-polygon intersection test is equivalent to the line-polygon one when considering only the geometries in $H_{f}$. Figure 10 also shows the challenging situation in which the vertices of a polygon may not belong entirely to one half-space. In this condition, the polygon shall be clipped into two sub-polygons, each one belonging to the corresponding half-space. For instance, with reference to Figure 10, triangle $T_{4}$ is split into the trapezoid $T_{4}^{f}$ and the triangle $T_{4}^{b}$. Only the trapezoid $T_{4}^{f}$ will be processed in the ray intersection test. This clipping procedure is explained in Section 5.3.1.

#### 5.3.1. Clipping

**Figure 11:**
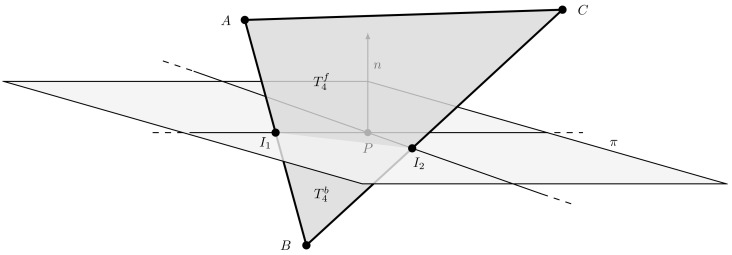
A triangle clipping example. The points $I_{1}$ and $I_{2}$ represent the intersections between the edges of the triangle ABC and the plane π. The trapezoid of vertices $AI_{1}I_{2}C$ is considered in front of the plane, while the triangle $I_{1}BI_{2}$ behind it.

The aim of this procedure is to determine the intersection points between the edges of the triangle and the plane that divides it. As we can see in Figure 11, to identify the polygons $T_{4}^{f}$ and $T_{4}^{b}$ in which the triangle is divided, we need to compute the intersection points $I_{1}$ and $I_{2}$ with the plane π.

**Figure 12:**
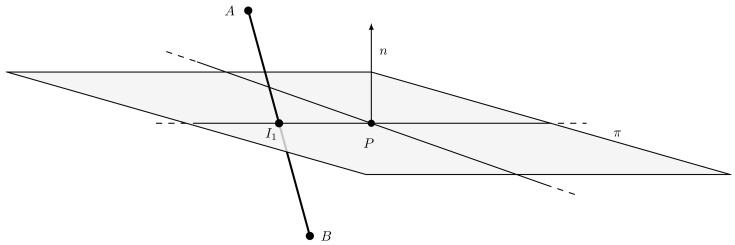
Segment clipping example. The point $I_{1}$ is the intersection between the segment AB and the plane π. The segment $AI_{1}$ is considered in front of the plane, while $BI_{1}$ behind it.

Consider the intersection point $I_{1}$. With reference to Figure 12, it is possible to classify the points *A* and *B* to be either in front, back or on the plane π defined by the normal *n* and a point *P*.

The classification can be done by looking at the signs of $d_{A}$ and $d_{B}$, which are the scalar products between *n* and the vectors A−P and B−P, respectively:

$$d_{A}=n^{T}\left(A-P\right)\;,\;d_{B}=n^{T}\left(B-P\right)\;.$$

If sign $(d_{A})>0$, the point *A* is in front of the plane while, if sign $(d_{A})<0$, on the back and, if $d_{A}=0$, the point lies on the plane. From those observations, it is clear that an intersection between the segment AB and the plane π is only possible if the following condition holds:

$$\mathrm{sign}\left(d_{A}\right)\neq \mathrm{sign}\left(d_{B}\right)\;\mathrm{OR}\;\mathrm{sign}\left(d_{A}\right)\cdot \mathrm{sign}\left(d_{B}\right)=0\;.$$

Note that the intersection point $I_{1}$ between segment AB and plane π can be expressed as

$$I_{1}=A+t\left(B-A\right),$$

where t∈[0,1]. It is possible to compute the value of *t* by observing that the vector $I_{1}-P$ lies on the plane and, therefore, $n^{T}\left(I_{1}-P\right)=0$. We obtain:

$$\begin{array}{cc} 0 & =n^{T}\left(I_{1}-P\right)=n^{T}\left(A-P\right)+t\;n^{T}\left(B-A\right)\\ \; & =n^{T}\left(A-P\right)+t\;n^{T}\left(B-P+P-A\right)\\ \; & =d_{A}+t\left(d_{B}-d_{A}\right)\;. \end{array}$$

This implies:

$$t=\frac{d_{A}}{d_{A}-d_{B}}\;.$$

Finally, we obtain:

$$I_{1}=A+\frac{d_{A}}{d_{A}-d_{B}}\left(B-A\right)\;.$$

In a similar way we can compute the intersection point $I_{2}$. By using the above procedure, it is possible to divide any polygon w.r.t. an arbitrary plane π in 3D space into its front and back geometries. For example, by assuming that the polygon has three vertices, the only case in which clipping is required is when a vertex belongs to a different half-space of the others. Without loss of generality, we assume that $B\in H_{b}$, while $A,C\in H_{f}$. The clipping procedure can be summarized with the following points:

- Compute intersection points $I_{1}$ and $I_{2}$ of segments AB and BC with the plane π using the procedure described above.
- Generate two sub-polygons: the triangle $I_{1}BI_{2}\in H_{b}$ and the trapezoid $AI_{1}I_{2}C\in H_{f}$.

Figure 11 shows the result of this procedure.

In our implementation, given a point $P_{j}$ on $T_{i}$ and a pencil of *K* rays starting from $P_{j}$, the clipping procedure is applied using the same plane for all the rays; specifically the plane used is the one containing the triangle $T_{i}$.

### 5.4. Proposed Approach Implementation and Its Convergence Properties

The implementation of the strategy we described in the previous sections, is reported in Algorithm 1.

**Algorithm 1** VSD based on Plücker coordinates
**Input**: 3D mesh $T=\{T_{1},\ldots ,T_{N}\}$ **Implementation**: For each $T_{i}$, the following actions are performed in order: select *M* points, $P_{1},\ldots ,P_{M}$, on $T_{i}$ (see Section 5.1);for each point $P_{h}$, h=1,…,M, generate *K* rays (see Section 5.2);for each of the MK rays generated at the previous point, perform the ray intersection test against all the triangles $T_{j}\neq T_{i}$ (see Section 5.3); if a ray intersects $T_{j}$, then it is removed from the set of generated rays;compute the visibility score of triangle $T_{i}$ as in (8). **Classification**: For $i\in \left\{1,\ldots ,N\right\}$, triangle $T_{i}$ is classified as visible is $SV_{\mathrm{score}}\left(T_{i}\right)>0$, otherwise as non visible.

Observe that, based on the proposed procedure, a non-visible triangle will be always classified as non visible, while a visible triangle might be erroneously classified as non-visible. However, it is possible to see that by increasing *M* and *K*, i.e., the number of points randomly selected on any triangle and the number of rays generated starting from any selected point, respectively, the misclassification error decreases. This fact is formally stated in the following propositions.

**Proposition** **1.** *Consider Algorithm 1. Assume that*

- *the M points in step (1) are selected either uniformly random or adopting the minimum distance approach; and*
- *the K rays in step (2) are generated uniformly random*.

*Then, for M and K going to infinity, that is*, M,K→∞, *the probability that a visible triangle is classified as non visible goes to* 0.

**Proof.**  Let S be a sphere that contains the entire mesh. Consider a visible triangle $T_{i}$. The fact that $T_{i}$ is visible means that there exists a point $Q_{1}$ on $T_{i}$ and a point $Q_{2}$ on S such that the segment connecting $Q_{1}$ to $Q_{2}$ does not intersect any other triangle of the mesh. Let $\ell_{Q_{1}Q_{2}}$ be the line passing through $Q_{1}$ and $Q_{2}$ and let $C_{\ell_{Q_{1}Q_{2}}}^{r}$ be the infinitely long cylinder of radius *r* having as axis the line $\ell_{Q_{1}Q_{2}}$. Accordingly, define

- $I_{1}^{r}$ to be the region obtained by intersecting $C_{\ell_{Q_{1}Q_{2}}}^{r}$ with $T_{i}$; and
- $I_{2}^{r}$ to be the region obtained by intersecting $C_{\ell_{Q_{1}Q_{2}}}^{r}$ with S and containing $Q_{2}$.

Notice that, for r>0, both $I_{1}^{r}$ and $I_{2}^{r}$ are connected regions of non-zero measure.

Since $T_{i}$ is visible, it follows that there exists $\bar{r}>0$ such that the portion of $C_{\ell_{Q_{1}Q_{2}}}^{\bar{r}}$ included between $I_{1}^{\bar{r}}$ and $I_{2}^{\bar{r}}$ does not intersect any triangle of the mesh; in turn, this fact is true also for any segment connecting a point in $I_{1}^{\bar{r}}$ to a point in $I_{2}^{\bar{r}}$.

Since *M* goes to *∞*, the probability of randomly picking a point $P_{j}$ on $T_{i}$ belonging to $I_{1}^{\bar{r}}$ goes to 1 (either using the uniform selection or the minimum distance approach). Moreover, since also *K* goes to *∞*, the probability of selecting a ray starting from a given point $P_{j}$ that crosses $I_{2}^{\bar{r}}$ goes to 1. Hence, for M,K,→∞, $T_{i}$ will be classified as visible with probability that goes to 1. This concludes the proof.  □

**Proposition** **2.** *Assume that*

- *the M points in step (1) are selected either uniformly random or adopting the minimum distance approach; and*
- *the K rays in step (2) are generated according to the Fibonacci lattice distribution*.

*Then, there exists*$\bar{K}>0$, *such that, if*$K\geq \bar{K}$ and M→∞, *then the probability that a visible triangle is classified as non visible goes to 0*.

**Proof.**  Consider a visible triangle $T_{i}$. Let $I_{1}^{\bar{r}}$ and $I_{2}^{\bar{r}}$ be as in the proof of Proposition 1. As observed previously, since *M* goes to *∞*, the probability of randomly picking a point $P_{j}$ on $T_{i}$ belonging to $I_{1}^{\bar{r}}$ goes to 1 (either using the uniform selection or the minimum distance approach). Moreover, adopting the Fibonacci lattice distribution, there exists ${\bar{K}}_{i}$, such that, if $K\geq {\bar{K}}_{i}$, then, for any point $P_{j}$ selected on $T_{i}$, Algorithm 1 will generate at least one ray starting from $P_{j}$ that crosses the region $I_{2}^{\bar{r}}$. This implies that, for $K\geq K_{i}$ and M→∞, triangle $T_{i}$ will be correctly classified with probability 1. To conclude the proof, it suffices to define

$$\begin{array}{c} \bar{K}:=\max\left\{K_{i}\;:\;T_{i}\;\mathrm{is}\;\mathrm{visible}\right\} \end{array}$$

 □

Based on Remark 1, we may provide a modified version of Algorithm 1 where step (1) can be simplified by considering only the barycentre of the triangle instead of sampling it. Specifically, step (1) can be substituted by the following step: (1’) Compute the barycenter of $T_{i}$. Clearly, Proposition 1 and Proposition 2 are not still valid when implementing step (1’) instead of step (1). However, in our numerical tests described in Section 6, we have implemented this modified version of Algorithm 1 and the obtained results show that this choice does not affect the overall result. This fact is not surprising since we have considered the typical case where the size of each triangle is very small as compared to the size of the entire mesh.

## 6. Results

This section presents a series of numerical tests to evaluate the proposed algorithm efficiency and robustness. First we compare the performance of the ray-triangle intersection test obtained using our Plücker space-based approach with the use of the Möller-Trumbore algorithm [11], that represents the state-of-the-art in this scenario. We then compare our solution with a state-of-the-art method, based on ambient occlusion estimate in the image space, which is implemented in MeshLab; see [10] for all the implementation details. To carry on all of these tests, we used a midrange PC equipped with an Intel i3-6100 CPU, 8 GB DDR4 RAM, and a AMD RX-480 4 GB GPU with 2304 stream processors. In particular, we implemented all the algorithms using high-level shading language (HLSL). This allows us to take full advantage of the GPU power which is made specifically for computer graphics tasks.

### 6.1. Intersection Algorithms Comparison

The algorithm described in Section 5 is heavily based on the intersection test between rays and triangles. Therefore, the computation complexity strongly depends on the optimization of this test. For that reason, we decide to compare the performance of Plücker-based ray-triangle intersection algorithm with the Möller-Trumbore one. To compare the two methods we designed a numerical test that can be summarized in the following steps.

1. Generate a set of triangles T randomly within a cubic volume of 1 m×1 m×1 m.
2. Create a set of points S by sampling the surface of a sphere, with radius 2 m, centred in the cubic volume, using the Fibonacci distribution.
3. For each point S∈S, generate a ray starting from the volume centre and passing through *S*, thus generating a set of rays R.
4. For each ray in R, check if it is occluded by at least one triangle in T.

**Figure 13:**
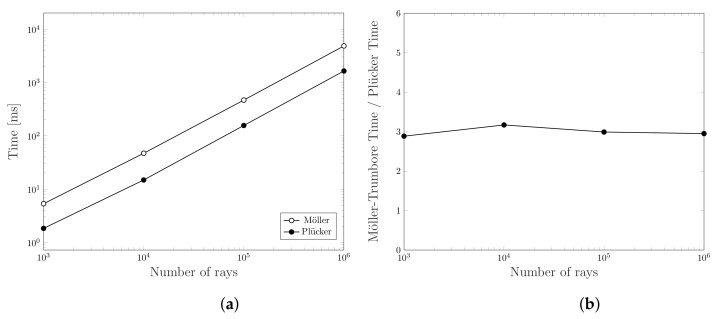
Computation time comparison for intersecting 10,000 triangles with an increasing number of rays using Möller-Trumbore and Plücker-based algorithms. (**a**) Computation time, (**b**) Ratio between the computation times of the two algorithms.

**Figure 14:**
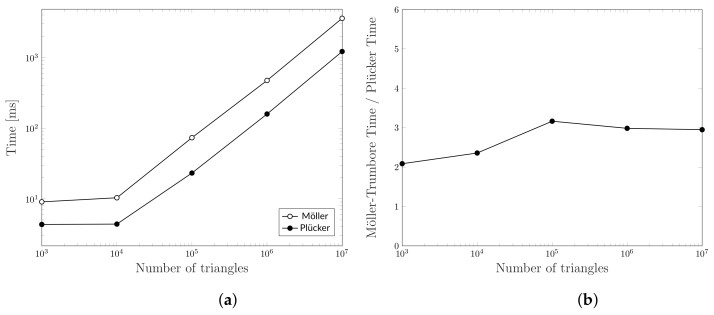
Computation time comparison for intersecting an increasing number of triangles with 1000 rays using Möller-Trumbore and Plücker-based algorithms. (**a**) Computation time, (**b**) Ratio between the computation times of the two algorithms.

Finally, the algorithm yields the following set:

V={r∈R:∃t∈T:intersects(r,t)}

where intersects(r,t) is either computed with Möller-Trumbore method or the Plücker-based one. Of course, the two methods produce the same set V but the number of operations they require is different. To provide a more reliable result, we reproduced all the tests 10 times by averaging the partial results. Moreover, since there are two parameters in the test algorithm, i.e., the number of sampled points in the sphere surface (|S|) and the number of generated triangles (|T|), we decide each time to fix one parameter and change the other to allow a more detailed and clear reading. Figure 13 shows the time spent to run the two algorithms and the ratio between them obtained using 10,000 triangles and a different number of rays. We have chosen this particular value because it allows all GPU cores to work full-throttle. Actually, Figure 14 shows that, by using a small number of triangles, some GPU cores remain in idle state. This figure, indeed, shows a performance comparison obtained using 1000 rays and a different number of triangles.

From the results, it is clear how Plücker-based algorithm always offers better performance than the Möller-Trumbore one in the cases tested. Indeed, the latter is about 3 times slower at the same load. An intuitive reason is related to the nature of the two algorithms. At each call of the intersection test, Möller-Trumbore must perform four dot products, three cross products, and three vectorial subtractions [11]. On the other hand, the method based on Plücker coordinates only needs 2 dot products and 1 vectorial subtraction. Although Plücker’s algorithm needs a challenging initialization, it allows to reuse some valuable information about the triangle that in Möller-Trumbore must be calculated on-the-fly at each iteration. Therefore, since we compare the same triangle with many different rays, the pre-processing done by Plücker’s algorithm allows us to save valuable time in the long run. Finally, we want to emphasize that Möller-Trumbore also computes the intersection point between a ray and a triangle. However, this information is totally irrelevant for our purposes.

### 6.2. Comparison with a State-of-the-Art Method

In this section, we compare the results obtained using the proposed algorithm with the ones provided by a state-of-the-art VSD method. The latter is based on the ambient occlusion estimate in image space and it is implemented within an open-source software called MeshLab [10]. MeshLab is widely used in computer graphics for processing and editing 3D triangular meshes. Among the many operations that this software offers, it provides visibility surface determination based on a custom version of the screen-space ambient occlusion (SSAO) [31]. This technique is typically used to improve the realism of a scene during rendering. Since it works in image space, it is characterized by a lower computational complexity. On the other hand, the dependence on the rendered image resolution limits the result quality. This limitation does not occur by estimating the ambient occlusion in object space using a ray tracing technique as presented in this work. The VSD method provided by MeshLab consists of the following steps.

1. Given a number of views *V*, *V* points are sampled on the surface of a sphere that fully embrace the triangular mesh using the Fibonacci distribution. The set R of camera directions contains all the rays starting from each sampled point and directed to the sphere origin.
2. For each direction r∈R, the depth map of the corresponding camera view is computed. By using this map the ambient occlusion term is computed for each pixel. Each value is then added to the correspondent projected triangle vertex.
3. For each vertex, the partial results obtained for each direction *r*, i.e., for each view, are averaged to obtain a unique global value.

After this process, it is possible to identify the invisible triangles by selecting those that have at least one occluded vertex of the three, i.e., with a zero ambient occlusion value. Obviously, the approximation improves as the number of views increases. With this brief description of the algorithm, that can be found at MeshLab GitHub repository [32], we want to highlight a couple of key points that distinguish the two approaches. As mentioned earlier, MeshLab VSD method works in the image space. This makes it significantly faster than the algorithm we propose but less robust, due to the dependence on the image resolution used when rendering depth maps. The second main limit regards the assumption that all triangles normals must be oriented towards the outside of the mesh. Actually, the method implemented in MeshLab is directly based on the notion of ambient occlusion presented in Section 4.1 and, therefore, it relies on triangle normals. The following numerical results show that, if triangles normals are flipped, the resulting estimate is totally meaningless. Actually, in the industrial domain, it may happen to observe models with some normals that are wrongly oriented. However, for the first tests, we have considered models with all the normal properly oriented.

**Figure 15:**
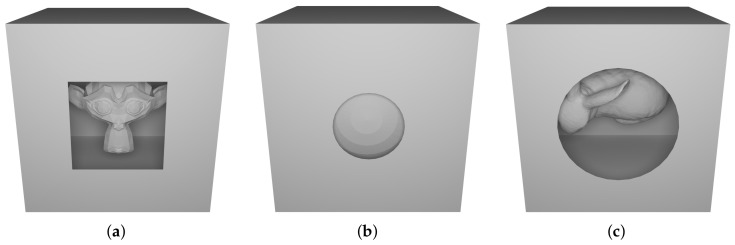
An overview of the 3D models used for numerical tests. (**a**) Sample 1 consists of 986 triangles, (**b**) Sample 2 of 1006 triangles and (**c**) Sample 3 of 9297 triangles.

To verify and compare performance in a reproducible way, we decided to create three different 3D models. As shown in Figure 15, a monkey head, a ball and a Stanford Bunny are respectively inserted in a box characterized by a hole in the front side. Since we want to test the ability to detect hidden triangles, we decided to design these ad-hoc models which perfectly fit an ideal scenario.

**Figure 16:**
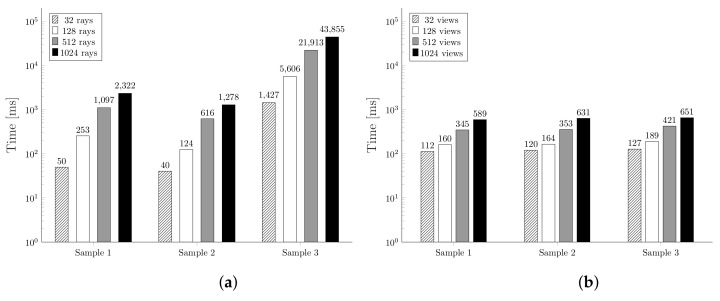
Computation time comparison of the two algorithms using different settings and samples. (**a**) shows the results obtained with Plücker VSD, (**b**) shows the results obtained with MeshLab VSD.

We start by comparing the time needed for the two algorithms to estimate the visibility for each face of the sample meshes. From Figure 16, we can notice how the results are fully expected due to the significant difference in complexity. Actually, we can infer that the computational complexity of MeshLab algorithm is O(VN), where *V* is the number of views and *N* the number of triangles. Indeed, the depth map calculation complexity is proportional to the number of triangles. As far as Algorithm 1 is concerned, we have implemented it by considering the modified step (1${}^{\prime }$) and the Fibonacci lattice distribution method; it can be seen that has a complexity of $O(KN^{2})$ where *K* is the number of rays tested for each triangle.

However, the time needed to perform VSD is not of particular interest for this work. In fact, recall that these algorithms are usually used in computer graphics only once to lighten 3D models. We aim for a robust method and, therefore, we are more interested in evaluating a performance index such as the number of incorrect classifications. Given the set of triangles T, we consider as misclassification error the sum of false positives and negatives divided by the number of triangles. Since we test if a triangle is invisible, false positives correspond to visible triangles that the algorithm identifies invisible. Consider the set $I_{A}$ of triangles classified as invisible by the algorithm and the set $I_{G}$ of truly invisible triangles which is known a priori. The misclassification error ME can be written as:

(9) $$\begin{array}{c} \mathrm{ME}=\frac{|\left\{t\in I_{A}:t\notin I_{G}\right\}\left|+\right|\left\{t\in I_{G}:t\notin I_{A}\right\}|}{\left|T\right|}\;. \end{array}$$

Notice that, as discussed in Section 5.4, false negatives cannot exist by construction of the algorithm, i.e., $|\{t\in I_{G}:t\notin I_{A}\}|=0$. Therefore, (9) can be simplified obtaining

$$\begin{array}{c} \mathrm{ME}=\frac{\left|\{t\in I_{A}:t\notin I_{G}\}\right|}{\left|T\right|}\;. \end{array}$$

We want to emphasize our interest in achieving the maximum lossless compression in terms of number of triangles. Since the maximum compression is reached when $I_{A}=I_{G}$, by minimizing ME we are maximizing the lossless compression.

**Figure 17:**
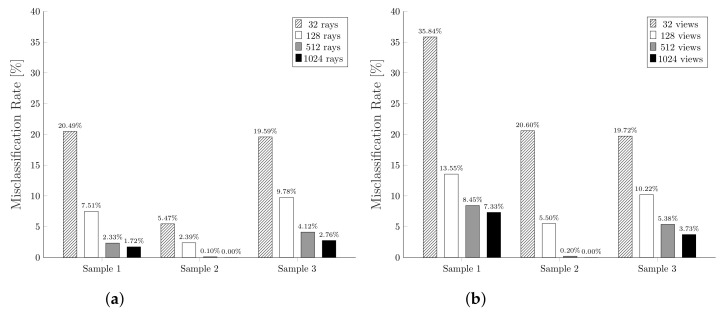
Misclassification rates comparison of the two algorithms using different settings and samples. (**a**) shows the results obtained with Plücker VSD, (**b**) shows the results obtained with MeshLab VSD.

Figure 17 shows the misclassification rates obtained using a different number of rays, or corresponding views, for each of the designed mesh samples. From these plots we can observe how, as the number of rays or views increases, the misclassification rate decreases. This is widely expected since, using fewer views or rays per triangle, it is more likely to misidentify a visible triangle as invisible and, thus, increase the number of false positives. Intuitively, having more views or rays per triangle allows the algorithm to find even the most hidden triangles, getting closer to the optimum value.

**Figure 18:**
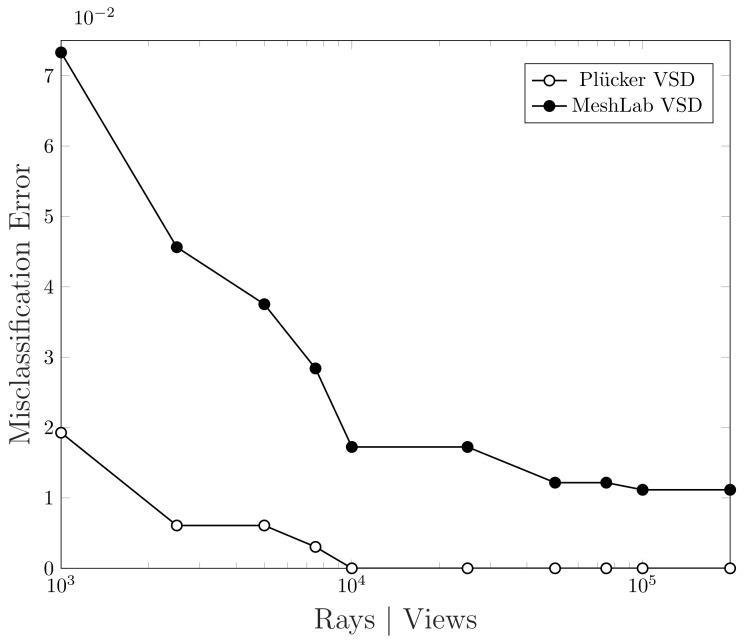
Convergence comparison of the misclassification error using the two algorithms on Sample 1.

From Figure 18 we can observe that our algorithm tends to the optimal faster than the method in comparison. Actually, as described previously, MeshLab VSD method operates in image space and it is constrained to a maximum resolution of 2048 × 2048 pixels [32]. As a result, even if the number of views increases infinitely, this method could never reach the optimal since sphere mapped points would have a finer quantization than the rendered image. This explains why the improvement in accuracy is so slow after a certain number of views. To be more precise, we report in Table 1 the evolution of the two misclassification rates with an increasing number of rays or views. In particular, we can notice that, using 10,000 rays, our algorithm is able to converge to the optimum while, MeshLab algorithm, stabilizes around 1% of error.

**Figure 19:**
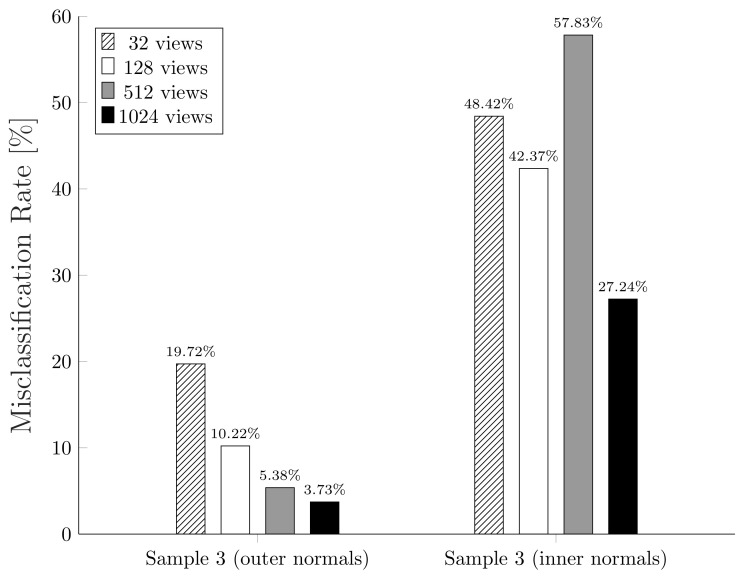
Misclassification rates comparison using MeshLab VSD algorithm on Sample 3 with outer and inner normals.

Finally, we want to highlight another significant result. At the beginning of this section, we stressed that one of the limitations of MeshLab algorithm regards the orientation of mesh triangles normals. Based on the notion of ambient occlusion, these normals must be oriented towards the outside of the mesh. This scenario is not so common in computer graphics: a triangular mesh may have normals flipped or it may not have normals at all. Indeed, as described in Section 3.1, depending upon the file format used to represent a mesh, there may or may not be encoded information on triangles normals. We then tried to flip all the normals of the third sample mesh and measuring the misclassification rate again as the number of views changed. Figure 19 shows how MeshLab algorithm is totally unable to solve the VSD problem in the case of incorrectly oriented normals. Our method, on the other hand, obtains the same results as before since it considers the entire sphere and not only the upper hemisphere according to the normal.

## 7. Conclusions

In this paper, we address the visibility problem in object space. We started from the notion of ambient occlusion and we adapted it to solve the VSD problem. Thanks to the use of Plücker coordinates, we were able to speed up the ray-triangle intersection test. We compared the performance of our algorithm against a state-of-the-art one which exploits another approach based on image space. This allowed us to evaluate pros and cons of a solution in object space with another in image space. Results showed that our approach is more robust in terms of convergence to the maximum lossless compression. In addition, it is resilient to normals orientation, a fundamental characteristic for the industrial context. Although the proposed solution is characterized by a high computational complexity, we stress that its impact is completely negligible since VSD techniques are typically used once per mesh. Anyway, there exist several acceleration techniques that can be adopted to speed up computations such as kd-trees, grids, bounding volume hierarchies [33]. Since the purpose of this work is to prove the result optimality, these improvements were not considered.

Future improvements may involve the creation of an hybrid approach. In particular, we could perform a first processing step with an image space VSD method and, then, a second step with our algorithm in order to give a more accurate result. In this case, our Plücker-based method will check only the invisible triangles recognized during the first step. This hybrid approach would allow to reduce the computational burden without decreasing the accuracy.

## Figures and Tables

**Table 1:** Detail of times and misclassification rates of VSD performed on Sample 1 by the two algorithms.

Rays|Views	MeshLab VSD		Plücker VSD	
	Time [s]	M. E. Rate [%]	Time [s]	M. E. Rate [%]
1000	0.58	6.39	2.29	1.93
2500	1.27	4.56	5.70	0.61
5000	2.41	3.75	11.39	0.61
7500	3.67	2.84	16.99	0.30
10,000	4.77	1.72	22.66	0.00
25,000	11.74	1.72	44.31	0.00
50,000	24.23	1.22	114.20	0.00
75,000	34.79	1.22	170.50	0.00
100,000	48.51	1.12	226.92	0.00
200,000	91.08	1.12	456.20	0.00

## Data Availability

No new data were created or analyzed in this study. Data sharing is not applicable to this article.
